# Assessment from Functional Perspectives: Using Sensorimotor Control in the Hand as an Outcome Indicator in the Surgical Treatment of Carpal Tunnel Syndrome

**DOI:** 10.1371/journal.pone.0128420

**Published:** 2015-06-08

**Authors:** Hsiu-Yun Hsu, Fong-Chin Su, Yao-Lung Kuo, I-Ming Jou, Haw-Yen Chiu, Li-Chieh Kuo

**Affiliations:** 1 Department of Physical Medicine and Rehabilitation, National Cheng Kung University Hospital, College of Medicine, 138 Shengli Rd., Tainan, 704, Taiwan; 2 Department of Biomedical Engineering, National Cheng Kung University, 1 University Rd., Tainan, 701, Taiwan; 3 Medical Device Innovation Center, National Cheng Kung University, 1 University Rd., Tainan, 701, Taiwan; 4 Section of Plastic Surgery, Department of Surgery, National Cheng Kung University, 1 University Rd., Tainan, 701, Taiwan; 5 Department of Orthopedics, National Cheng Kung University, 1 University Rd., Tainan, 701, Taiwan; 6 Department of Occupational Therapy, National Cheng Kung University, 1 University Rd., Tainan, 701, Taiwan; University of Ottawa, CANADA

## Abstract

To investigate whether sensorimotor control of the hand could be an outcome indicator after carpal tunnel release (CTR), this work examined changes in the results of patients’ manual tactile test (MTT), pinch-holding-up activity (PHUA), two-point discrimination (2PD) and Semmes-Weinstein monofilament (SWM) tests. Participants included 30 predominantly sensory neuropathy CTS patients, as confirmed by a nerve conduction study. The MTT, precision pinch performance in PHUA and traditional sensibility (2PD and SWM) tests were used to examine different aspects of sensory status at the time-points of two weeks before operation and one month post-operation, with a single-blind design. The results showed significant improvements in the sensory function as detected by the 2PD and SWM tests (*p*<0.001) and sensorimotor function as detected by the MTT (*p*<0.001) and PHUA test (*p*<0.05) for patients receiving CTR. The responsiveness of the SWM, MTT and PHUA tests (effect size>0.5, *p*<0.01) are better than that of two-point discrimination test (effect size<0.5, *p*<0.001). However, pinch strength saw a decline compared to baseline with a moderate effect sizes (effect size = 0.7, *p*<0.001). This cohort study found that the MTT and PHUA test can both meet all the statistical criteria with regard to assessing treatment outcomes for patients with CTS. In addition, the results of this work provide clinicians with the information that the sensorimotor functions of the hands, as assessed by MTT and PHUA, are responsive to clinical changes due to CTR.

## Introduction

Carpal tunnel syndrome (CTS) is a condition with a high prevalence worldwide [1, 2], and has been shown to negatively influence the health-related quality of life [3] and sensorimotor functioning [4] of patients. Clinically, CTS is caused by median nerve entrapment due to tunnel pressure being elevated at the wrist, [2, 5] and thus the best treatment is to relieve this pressure [6].

For early CTS, the combined use of the Semmes-Weinstein monofilament (SWM) test and nerve conduction studies (NCS) has been shown to provide the objective outcomes needed to identify sensory deficits in nerve compression disorders [7]. However, previous studies have shown that precision grip performance might be significantly affected in CTS patients, even if they only have sensory deficits without significant motor involvement [8, 9]. Moreover, the stimulus is given in a very subjective way when executing traditional tests (i.e., 2PD and SWM), and thus these are seen as very subjective measurements,[10] with more objective methods being needed in clinical practice to directly estimate the sensorimotor function with regard to the patient's hand capabilities [11].

Some recent studies revealed that people can recognize the properties of objects more clearly and precisely through a general grasping and a precise exploratory process [12–15]. To achieve this, a new manual tactile test (MTT) was developed to evaluate the integrated ability of perception with regard to object characteristics and functional hand uses [16]. In addition, the ability to adjust the hand pinch force according to the inertial load of the object being handled has been shown to be an objective evaluation of hand performance,[17–20], and one which correlates strongly with hand functioning [21].

Sensibility and muscle strength tests are frequently used to analyze how well CTS patients respond to treatment [22, 23]. However, the improvements in the delicate sensorimotor control of the hand have received relatively little attention. Clinically, the instruments for documenting treatment outcomes for nerve injuries are required to be valid and able to capture valuable information [24]. The PHUA and MTT can assess subtle changes in the sensorimotor abilities of the hand among patients with various degrees of CTS severity, diagnosed based on the results of electrophysiological studies [25, 26]. However, the responsiveness of these two new sensibility tests with regard to CTS treatment has never been analyzed. This study hypothesized that the MTT and PHUA are responsive enough to detect any improvements in the tactile capability and sensorimotor control of the hand, respectively, after CTR. Therefore, the specific aim of this study was to examine the early outcome changes in MTT, precision pinch performance in PHUA, traditional sensory tests and pinch strength tests for patients who received CTR to determine the potential applicability of these tools in clinical practice.

## Materials and Methods

### Patients

**Fig 1:**
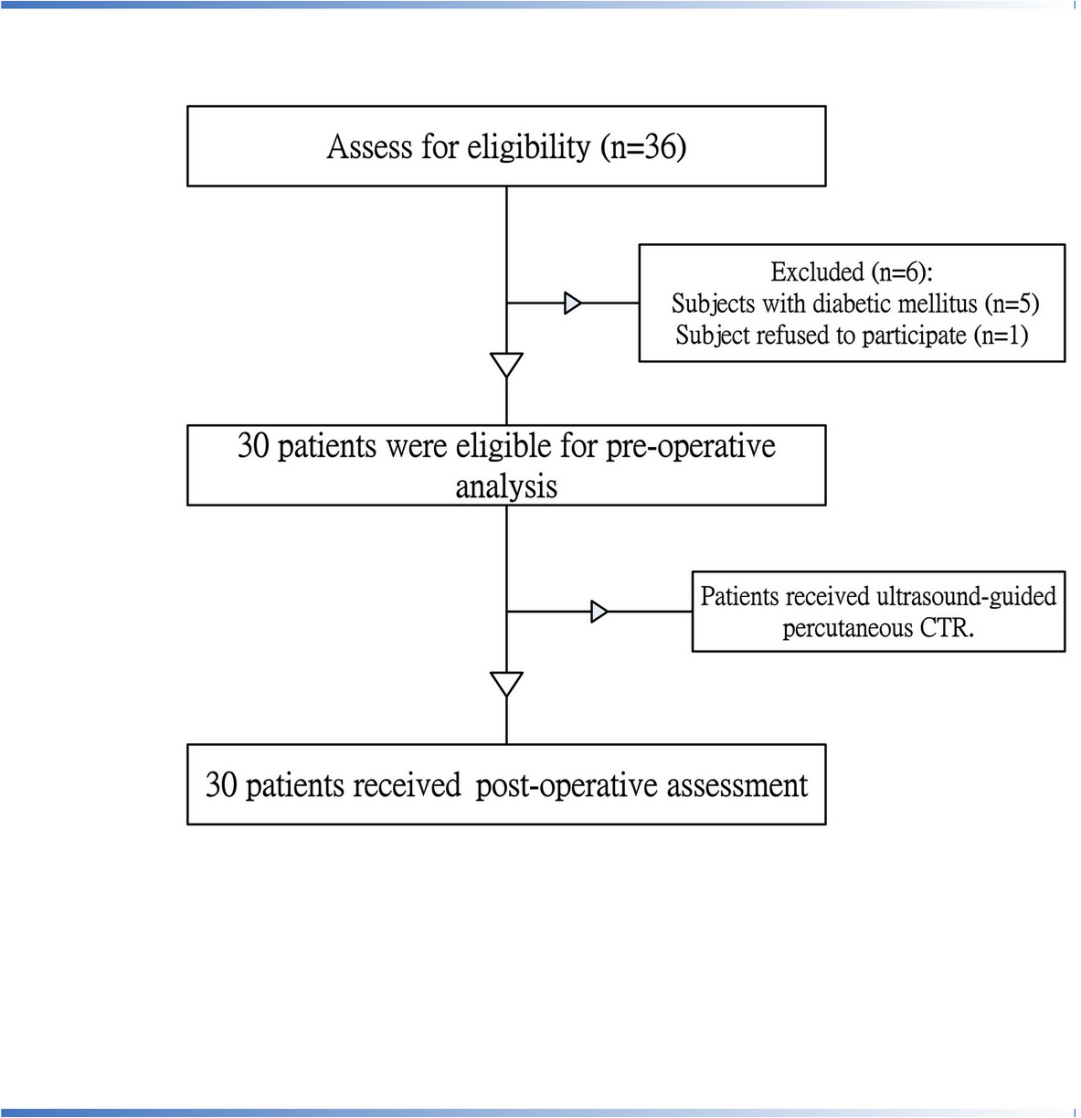
Flow chart shows enrollment of patients and completion of study.

This prospective cohort study recruited 30 CTS patients (Fig 1), as confirmed by a nerve conduction study, all of whom had predominantly sensory neuropathy. The enrolled patients received ultrasound-guided percutaneous CTR by an orthopedic surgeon of the Department of Orthopedics at a university hospital in southern Taiwan. The inclusion criteria for the patients were as follows: (1) significant sensory symptoms of CTS; (2) lesions involving the distal median nerve, as found by the electrophysiological test using a Medelec Synergy N-EP EMG apparatus (Oxford Instruments Medical, Inc., Tubney Woods, Abingdon, Oxon, UK). A peak latency of greater than 3.2 ms in this sensory nerve conduction velocity test is the main criteria to determine CTS [27]; and (3) with good-to-normal grade of muscle power of the abductor pollicis brevis muscle, as assessed by manual muscle testing. The exclusion criteria for the patients were those subjects with diabetic mellitus, other neurological deficits or previous hand injuries. An occupational therapist, a blinded assessor, conducted all the assessments at each of the time points in order to determine the early results with regard to the sensorimotor function of the hand.

### Ethics Statement

All participants were informed about the purpose of the study and signed consent forms. The study was approved by the Institutional Review Board (No. ER-98-257) of National Cheng Kung University Hospital.

### Design

This study had a single-blind design with pre- and post-operative assessments. When a new eligible participant was enrolled, a number of examinations were conducted before and after CTR, including traditional sensibility tests, PHUA tests, MTT and maximum pinch strength tests. The sequence of testing was randomized to minimize potential order effects. The time-points of evaluation were set at two weeks pre-operation, and one month post-operation.

### Instruments

#### Manual tactile test (MTT)

The barognosis, roughness differentiation and stereognosis subtests were included in the MTT, which were designed to assess the abilities to discriminate the weight, texture and shape properties of objects, respectively. Weight perception was tested with three cylinders of the same size and shape, using three different weights: 150g, 225g and 300g. Texture was tested with plastic cubes (1-inch in width) of the same weight and shape, and with smooth, rough and roughest surfaces. Six objects were used for each texture perception, giving 18 cubes in total. Shape perception was tested with three different geometric-shaped objects, cubes, ellipsoids and spheroids, which were made of plastic and had the same weight and roughness. Each configuration again consisted of six objects, with 18 objects in total. Each subtest of the MTT was conducted with the following standardized procedures. *(1) Barognosis subtest*: Three cylinders with different weights were placed in a line in a random sequence in front of the subject. Each subject was instructed to use their right hand to pick up the cylinder located on their right side (or the left side if their left hand was being examined) and put it in front of the original location. After moving all three cylinders, the subject had to point to the heaviest one. *(2) Roughness differentiation subtest*: During the testing procedures, a curtain was placed in front of the subject to block their view. Two boxes (25 cm × 15 cm × 4 cm) were placed in front of the curtain so that the subject could not see the cubes inside the right box (left box for left hand). Eighteen cubes were randomly placed inside the box. The subject was then informed to pick up any cube and to feel the texture. If the subject felt the roughest one, they were instructed to place it in front of the right box (left box for left hand). Otherwise this cube should be placed inside the left empty box (right empty box for left hand). The test was completed when the last cube was correctly placed. *(3) Stereognosis subtests*: The setup, procedures and scoring for this subtest were similar to those in the roughness differentiation test. The subject was informed to pick up any object and feel its shape. If the subject felt the shape to be spherical, they were instructed to place it in front of the right box. The time required from picking up the first object to the final placement of the last object was recorded. For each test, the dominant hand was tested first. All subjects were asked to perform the test as quickly as possible. The test procedures were repeated three times for each hand, with a one-minute resting interval between trials. The examiner made sure that the subject picked up an object each time they needed to. The time needed to complete each test was recorded using a stopwatch.

#### Pinch-holding-up-activity (PHUA)

**Fig 2:**
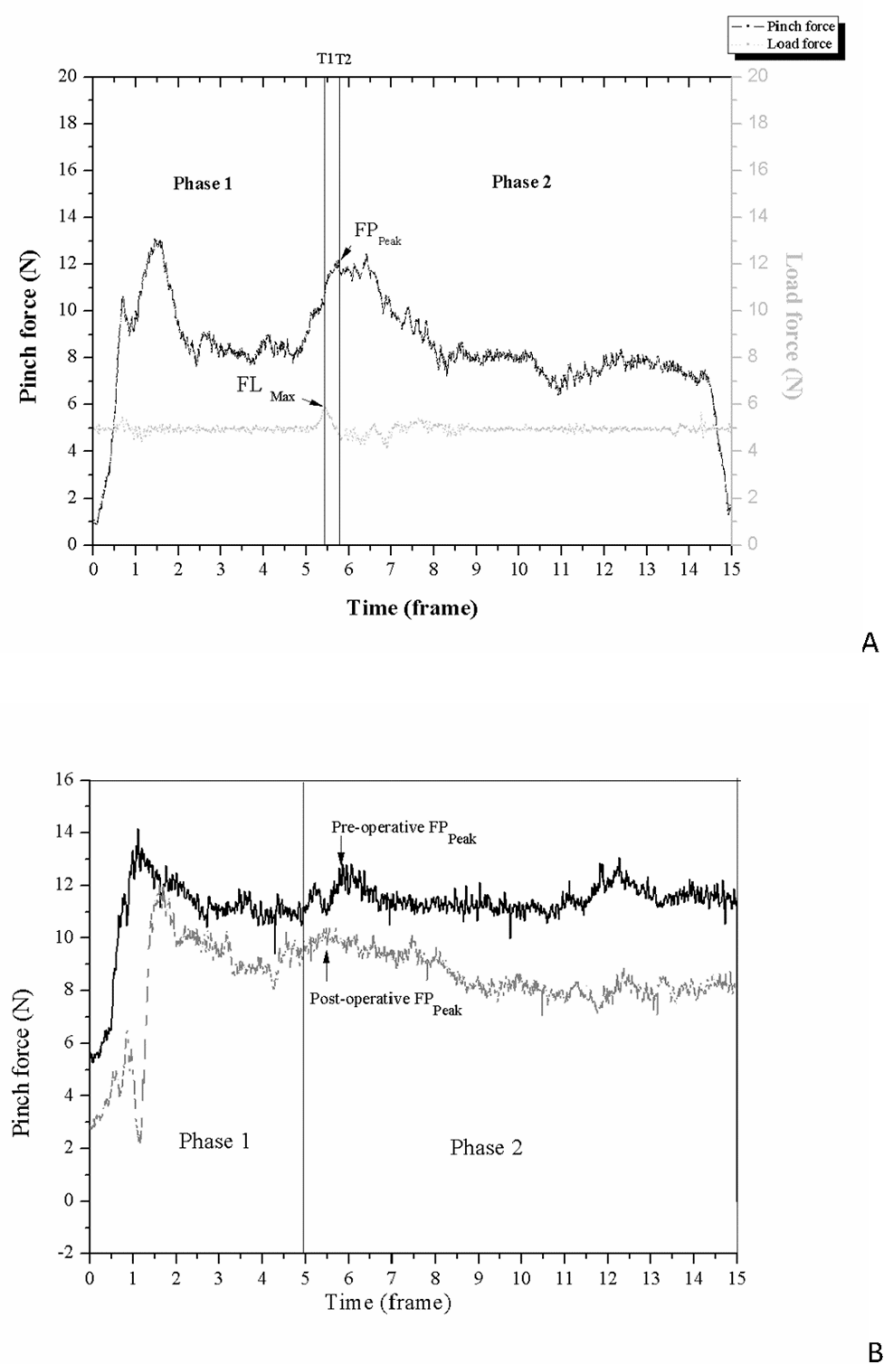
Pinch force and load force in the force–time relationship figure. (A) The peak pinch force (FP_Peak_) and the maximum load force (FL_Max_) are shown. (B) The pinch force with time series detected at the pre- and post-operative assessments of subject 6.

The pinch apparatus (weight = 480g) was designed to detect the subjects’ precision pinch performance. A six-axis load cell (Nano-25; ATI Industrial Automation, Apex, NC) and an accelerometer (Model 2412; Silicon Designs, Inc., Issaquah, WA) were embedded in a cuboid-device to detect the pinch forces exerted by the thumb and index finger and the acceleration of the pinch apparatus in space, respectively. The load of the pinch apparatus was computed as the product of mass (m) and the vector summation of gravity (g) and the lifting acceleration (a) detected by the accelerometer. Before the data collection, the subjects were instructed regarding the timing of task sequence. The procedures of the PHUA test were as follows: In the first phase, the subject pinched and lifted the apparatus with the pulps of thumb and index finger to 5 cm above the table using the guided target, and held this position for 5 seconds. In the second phase, the subjects were then asked to lift the apparatus to a height of 30 cm with a self-paced lifting speed, and then slowly lower it to its initial position. The data collection period for each trial was 15 seconds (Fig 2A). The maximum load force (FL_Max_) of the apparatus (T1) and the peak pinch force (FP_Peak_) (T2) during phase 2 were recorded. The force ratio between FP_Peak_ and FL_Max_ was used to determine the capacity for adjusting pinch force according to inertial load fluctuations, and this had a high testing reliability [28]. A score of 2.66 for force ratio is the optimal cutoff point when screening for CTS patients [25]. In addition, the time lag between T1 and T2 were analyzed to understand the precise temporal coupling between grip and load force profiles after CTR. After three trials, the subjects performed a pulp pinch with their maximal force to record the static maximal pinch force. Moreover, the peak pinch force divided by the maximal static pinch strength was also computed as a percent of maximum voluntary contraction.

#### Traditional Sensibility Tests

The two-point discrimination test is widely used in clinical settings to measure tactile spatial acuity. The static and moving two-point discrimination (S2PD and M2PD) tests were used to evaluate the innervated density of slowly and quickly adapting mechanoreceptors, respectively. During testing, one or two prongs of the discriminator (Ali Med, Dedham, MA) were applied randomly to the digital pulp to determine the least distance that the subject could detect [11]. The SWM test using Touch Test Sensory Evaluators (North Coast Medical, Inc, Gilroy, CA) examines the cutaneous pressure threshold by using a filament that exerts a constant force onto the palmar skin area for 1–1.5 seconds. The threshold was defined as the lightest filament that the subject responses correctly at least two out of the three trials. The filaments are labeled with a numerical marking, which is a log to the base ten of the force in tenths of milligrams [29]. The pulps of thumb and index finger were assessed and the mean value of the two digits for each tests were calculated as the final score.

#### Clinical Global Impression Scale- Improvement Item (CGI-I)

The CGI-I [30] was administered to the patients at the post-operative visit to assess any self-reported improvements over the treatment period. The scale, rated from 1 (very much improved) to 7 (very much worse), is as the indicator for determining any changes perceived by the patients following CTR treatment. However, due to the small sample size in the current study, the CGI-I was dichotomized into ‘improved’ and ‘not improved’ categories (Table 1).

**Table 1:** Self-perceived improvement on the CGI—I scale for the CTR (n, %).

CGI-I scale		Aggregated CGI-I category
Very much improved	5 (16.7)	
Much improved	12 (40)	Improved 23 (76.7)
Minimally improved	6 (20)	
Neither improved nor worse	2 (6.7)	
Minimally worse	3 (10)	Not improved 7 (23.3)
Much worse	1 (3.3)	
Very much worse	1 (3.3)	

n: indicates the number of patients.

### Data Analysis

The discriminative sensation was assessed by determining the minimum distance which the subjects could detect in the static and moving 2PD tests. The numerical marking determined from the SW monofilament test was defined as the pressure threshold of the hands. The force ratio and time lag between FP_Peak_ and FL_Max_ were used in the analysis of the PHUA task. For the MTT, the average time required to perform each subtest for three trials was calculated as the final scores.

### Statistical Analysis

The statistical analysis of this study was performed using SPSS 17.0 for Windows (Statistical Package for Social Sciences Inc. Chicago, IL, USA). The descriptive statistics were used to describe the means and standard deviations of the dependent variables, including the results of demographic data, 2PD, SWM, the MTT and PHUA tests. Paired *t*-tests were used to test the differences between the results for the patients before and after surgery. Effect size statistics (mean change divided by the pre-operative standard deviation) was used to measure the responsiveness of each test [31]. The effect size (ES) is graded as small (ES range, 0.2–0.5), moderate (ES range, 0.5–0.8) and large (ES >0.8) [32]. The statistical power was examined using the G*Power 3.0.10 software [33]. The minimal clinically important difference (MCID) for the 2PD, SWM, MTT and PHUA tests was determined by calculating the mean difference and 95% confidence interval (CI) of the change between the pre- and post-operative results for the patients who reported ‘improved’ and ‘not improved’ following CTR treatment. The level of significance was set at *p*≤0.05.

## Results

The results of the MTT, PHUA, maximum static pinch strength and traditional sensibility tests for the subjects before and after CTR are shown in Table 2. The subjects had better discriminative and threshold sensibility, as assessed by the from S2PD, M2PD, and SWM tests (*p*< 0.001), following CTR. The time-requirement of the three subtests of the MTT meant that the CTS patients showed significant differences before and after CTR (*p*<0.001). The values of the barognosis subtest ranged from 3.40±0.98 s pre-operatively to 2.81±0.75 s post-operatively, while the results of the roughness differentiation subtest ranged from 49.37± 19.93 s to 38.16±14.71 s, and those for the stereognosis subtest from 36.49±9.78 s to 30.91±8.46 s. The patients were also faster and more efficient with regard to force scaling when executing PHUA after CTR (Fig 2B; Table 2). The values of the force ratio were 2.93±0.37 and 2.54±0.16 in the pre-operative and post-operative assessments, respectively, (*p*<0.001). The time lag between the FP_Peak_ and FL_Max_ also revealed a statistically significant difference between pre- and post-operative evaluations (29±16 ms and 20±15 ms respectively, *p* = 0.009). However, the maximum static pinch strength decreased significantly, from 38.6±9.2 N to 32.2±8.2 N, after CTR (ES = 0.7, *p*<0.001). A small ES was observed for the 2PD test, while a moderate ES was found for the SWM, MTT and pinch strength tests. The force ratio part of the PHUA was the most responsive test, with a large ES before and after CTR (Table 2). The statistical power for detecting the difference among the pre-operative and post-operative tests was greater than 0.9 for the traditional tests (S2PD, M2PD, and SWM), barognosis, roughness differentiation and stereognosis subtests of the MTT, and also for the parameter of the force ratio detected by the PHUA test. Nevertheless, the power was only 0.78 for the parameter of time lag of the PHUA test.

**Table 2:** Differences in sensory and motor function before and after carpal tunnel release (n = 30).

	Pre-operative assessment (Mean ± SD)	Post-operative assessment (Mean ± SD)	*p*-value	Effect size
**Traditional sensibility test**				
S-2PD (mm)	5.8± 3.1	4.6±3.1	<0.001	0.39
M-2PD (mm)	5.0±3.2	3.6±2.4	<0.001	0.44
Semmes-Weinstein test	3.59±0.41	3.28±0.44	<0.001	0.76
**Manual tactile test**				
Barognosis test	3.40±0.98	2.81±0.75	<0.001	0.60
Roughness differentiation test	49.37± 19.93	38.16±14.71	<0.001	0.56
Stereognosis test	36.49±9.78	30.91±8.46	<0.001	0.57
**PHUA test**				
Force ratio	2.93±0.37	2.54±0.16	<0.001	1.05
Time lag (ms)	29±16	20±15	0.009	0.56
**Maximal static pinch strength (N)**	38.6±9.2	32.2± 8.2	<0.001	0.70
**Nerve conduction study**				
Peak distal latency (m secs)	4.17 ± 0.85			
Conduction velocity (m/ sec)	29.45 ± 6.57			
Amplitude (μV)	8.90 ±6.20			

S-2PD: static two-point-discrimination; M-2PD: moving two-point-discrimination; n indicates the number of hands. Statistics: Paired-*t* test; the significance level was set at 0.05.

Of the recruited subjects, seven of them reported ‘not improved’ and twenty-three reported ‘improved’ following CTR based on their assessments of the GCI-I category (Table 1. Regarding the anchor-based MCID, the differences in the changes in values in the SWM, S2PD, and M2PD between the ‘not improved’ and ‘improved’ groups were 0.70 (95% CI = -0.25–0.39, *p* = 0.66), 1.00 (95% CI = -0.90–2.90, *p* = 0.29) and 0.75 (95% CI = -1.17–2.67, *p* = 0.43), respectively. In addition, the differences in changes in values in the barognosis, roughness differentiation and stereognosis subtests between the two groups were 0.28 (95% CI = -0.75–0.19, *p* = 0.24), 1.00 (95% CI = -10.20–8.19, *p* = 0.83) and 0.99 (95% CI = -5.66–3.69, *p* = 0.67) seconds, respectively, between the two groups. In addition, the differences in the changes in values between the ‘not improved’ and ‘improved’ group following CTR were 0.31 *(*95% CI = 0.06–0.57, *p* = 0.018*) for the force ratio and 1.69 (95% CI = 0.56–2.83, *p* = 0.005*) for the FP_Peak_.

## Discussion

This study investigated the responsiveness of the MTT, PHUA and traditional sensory tests for CTS patients who received CTR. With the exception of the 2PD test, the effect sizes of the SWM, MTT and PHUA tests were moderate to large at one month post-surgery. In agreement with the results of recent research [22, 34], SWM and tactile gnosis are more responsive tests than the 2PD test. The CTS patients had statistically significant improvements in both sensory status and sensorimotor control of the hands (*p*<0.001); in contrast, the muscle strength of the pincer grip decreased after CTR (Table 2). The subjects only regained 83.4% of their pre-operative levels of grasp strength four weeks after their operations, similar to the findings in Gellman [7]. Clinically, the dissection of the transverse carpal ligament during CTR might contribute to pillar pain, and then lead to inadequate recovery of pinch force at the early follow-up stage for patients who have received CTR [35].

The integrated impulses transmitted both from the proprioceptors and mechanoreceptors help the hands perceive sensory information more accurately and rapidly [36]. The MTT was developed to detect perceptions regarding the weight, roughness, and geometric characteristics of an object from skin contact integrated with kinesthetic information from the movement of the hand, and has been reported to be a valid and assessment tool for hand sensibility, with good repeatability [26, 37]. Compared to the cut-off values for diagnosing CTS in the barognosis, roughness, and stereognosis tests (2.66s, 33.04s and 28.05s respectively) [37], the subjects in this study had deficits in their perceptions of the weight, texture and shape properties of objects. The significant improvements seen in the results of MTT carried out in this study for the patients who had received CTR indicate that this new method can meet all the statistical criteria for an examination tool with regard to detecting such sensory improvements. As the high measuring consistency has been reported for the MTT [37], there is no learning effect with regard to reducing the time-requirement in this cohort study. Although the follow-up period was relatively short in the current work, the improved abilities to identify weight, shape and roughness among the CTS patients show that they had the potential to make significant progress after CTR, as seen in the findings of previous research [22]. Though the 2PD test has been routinely used clinically, a previous report has indicated its invalid to reflect the tactile spatial acuity of hands [38]. Therefore, the results of MTT could thus be used as an augmented index for detecting discriminating sensation in the hands of patients who have been treated for CTS.

A functional repertoire model has been proposed as a framework to guide assessment and therapy of hand injuries [39]. Precision pinch force is used in an experimental model to investigate how cutaneous afferents contribute to motor performance [40]. In addition, precision pinch performance has been found to be significantly correlated to hand function [21]. A few recent studies noted that sensory disturbances can compromise the motor functioning of CTS patients [9, 41]. In the current study, the results of force scaling and temporal coupling with regard to the grip and load force profiles (Fig 2B) improved to a statistically significant degree following surgery, the same as was found for the discriminative sensibility tests. Based on Fitts’ skill acquisition model [42], motor performance is based on the human motor capacity integrated with related sensory feedback; and thus improved sensory functioning can help to restore precision pinch performance, as found in previous studies [43, 44]. Moreover, a statistically significant difference was been found in the change in values of the force ratio *(*95% CI = 0.06–0.57, *p* = 0.018*) and FP_Peak_ (95% CI = 0.56–2.83, *p* = 0.005*) between the ‘not improved’ and ‘improved’ groups, but not in the MTT and traditional sensibility tests. The results with regard to the MCID suggest that the patients’ perceptions have some clinical value, and thus that assessment of sensorimotor control of the hands by examining functional performance is also useful.

Different from the traditional sensory evaluations, which focus on submodality-specific perceptions, the PHUA and MTT are used to assess the abilities of sensorimotor control and actively sensing weight, geometry and texture for patients with impaired sensibility. In agreement with previous studies [25, 26], the MTT, precision pinch performance, and traditional sensibility tests are all valid tools with high statistical power for detecting differences in hand sensibility from various perspectives. In fact, this work is a pilot study to verify the responsiveness of the MTT and PHUA test with regard to the outcomes of CTS treatment. The results show that these new tests meet the requirements of hand sensibility evaluation, and could be used in conjunction with other tools to obtain a comprehensive assessment of the related sensorimotor deficits. Clinically, the focus of treatment for carpal tunnel syndrome is to relieve symptoms and allow the patient to return to normal hand functions. Using the sensorimotor tests can provide valuable information that can objectively identify the changes in hand functional performances due to the CTS treatments. In addition, unlike the costly and time-consuming nerve conduction study, the efficient and cost-effective characteristics support the clinical utility of these novel assessment tools. In the future, the potential application of these measurements could be investigated through establishing the relationship between hand dexterity and different degrees of severity in CTS. However, there are some limitations in this study that should be noted. First, the measuring apparatus required for such a sophisticated precision pinch performance assessment is not readily available in most clinical settings. Moreover, the MTT and PHUA test both require motor functioning, and thus they have little ability to assess postoperative improvements among advanced CTS patients with impaired motor capacity. Further work should refine the testing apparatus in order to increase its availability and applicability in most clinical settings. In addition, the grating orientation task has been proposed as a valid and objective tool for assessing spatial acuity of hand sensation, therefore, the relationship between the two novel tests and grating orientation task will be worthy to be analyzed in the future [45, 46].

## References

[1] Atroshi I, Gummesson C, Johnsson R, Ornstein E, Ranstam J, Rosen I. Prevalence of carpal tunnel syndrome in a general population. JAMA. 1999;282(2):153–8. Epub 1999/07/20. joc81321 [pii]. . 10411196 10.1001/jama.282.2.153

[2] Kremer M. Diagnosis of the carpal tunnel syndrome. Lancet. 1985;1(8433):854–5. Epub 1985/04/13. S0140-6736(85)92213-5 [pii]. . 2580200

[3] Atroshi I, Gummesson C, Johnsson R, Sprinchorn A. Symptoms, disability, and quality of life in patients with carpal tunnel syndrome. J Hand Surg Am. 1999;24(2):398–404. Epub 1999/04/08. S0363502399000726 [pii]. . 10194028 10.1016/s0363-5023(99)70014-6

[4] Barr AE, Barbe MF, Clark BD. Work-related musculoskeletal disorders of the hand and wrist: epidemiology, pathophysiology, and sensorimotor changes. J Orthop Sports Phys Ther. 2004;34(10):610–27. Epub 2004/11/24. 10.2519/jospt.2004.34.10.610 15552707 PMC1557630

[5] Keith MW, Masear V, Chung K, Maupin K, Andary M, Amadio PC, et al Diagnosis of carpal tunnel syndrome. J Am Acad Orthop Surg. 2009;17(6):389–96. Epub 2009/05/29. 17/6/389 [pii]. . 19474448 10.5435/00124635-200906000-00007 PMC5175465

[6] Manktelow RT, Binhammer P, Tomat LR, Bril V, Szalai JP. Carpal tunnel syndrome: cross-sectional and outcome study in Ontario workers. J Hand Surg Am. 2004;29(2):307–17. Epub 2004/03/27. doi: 10.1016/j.jhsa.2003.11.001 S0363502303006415 [pii]. . 15043907 10.1016/j.jhsa.2003.11.001

[7] Gelberman RH, Szabo RM, Williamson RV, Hargens AR, Yaru NC, Minteer-Convery MA. Tissue pressure threshold for peripheral nerve viability. Clin Orthop Relat Res. 1983;(178):285–91. Epub 1983/09/01. . 6883862

[8] Jeng OJ, Radwin RG, Rodriquez AA. Functional psychomotor deficits associated with carpal tunnel syndrome. Ergonomics. 1994;37(6):1055–69. Epub 1994/06/01. 10.1080/00140139408963718 . 8026451

[9] Tamburin S, Cacciatori C, Marani S, Zanette G. Pain and motor function in carpal tunnel syndrome: a clinical, neurophysiological and psychophysical study. J Neurol. 2008;255(11):1636–43. Epub 2008/08/05. 10.1007/s00415-008-0895-6 . 18677642

[10] Chapman CE. Active versus passive touch: factors influencing the transmission of somatosensory signals to primary somatosensory cortex. Can J Physiol Pharmacol. 1994;72(5):558–70. Epub 1994/05/01. . 7954086 10.1139/y94-080

[11] Dellon AL. Evaluation of sensibility and re-education of sensation in the hand. Baltimore: Williams and Wilkins; 1981.

[12] Naito E, Ehrsson HH. Somatic sensation of hand-object interactive movement is associated with activity in the left inferior parietal cortex. J Neurosci. 2006;26(14):3783–90. Epub 2006/04/07. 26/14/3783 [pii] 10.1523/JNEUROSCI.4835-05.2006 . 16597731 PMC6674143

[13] Overvliet KE, Smeets JB, Brenner E. The use of proprioception and tactile information in haptic search. Acta Psychol (Amst). 2008;129(1):83–90. Epub 2008/06/20. S0001-6918(08)00062-0 [pii] 10.1016/j.actpsy.2008.04.011 . 18561891

[14] Smith AM, Chapman CE, Donati F, Fortier-Poisson P, Hayward V. Perception of simulated local shapes using active and passive touch. J Neurophysiol. 2009;102(6):3519–29. Epub 2009/10/16. 00043.2009 [pii] 10.1152/jn.00043.2009 . 19828730

[15] Williams PS, Basso DM, Case-Smith J, Nichols-Larsen DS. Development of the Hand Active Sensation Test: reliability and validity. Arch Phys Med Rehabil. 2006;87(11):1471–7. Epub 2006/11/07. S0003-9993(06)00974-9 [pii] 10.1016/j.apmr.2006.08.019 . 17084122

[16] Hsu HY, Kuo LC, Jou IM, Chen SM, Chiu HY, Su FC. Establishment of a Proper Manual Tactile Test for Hands With Sensory Deficits. Arch Phys Med Rehabil. 2012. Epub 2012/08/14. S0003-9993(12)00594-1 [pii] 10.1016/j.apmr.2012.07.024 . 22885285

[17] Cole KJ, Steyers CM, Graybill EK. The effects of graded compression of the median nerve in the carpal canal on grip force. Exp Brain Res. 2003;148(2):150–7. . 12520402 10.1007/s00221-002-1283-6

[18] Duff SV. Impact of peripheral nerve injury on sensorimotor control. J Hand Ther. 2005;18(2):277–91. Epub 2005/05/14. S0894113005000475 [pii] 10.1197/j.jht.2005.02.007 . 15891985

[19] Lowe BD, Freivalds A. Effect of carpal tunnel syndrome on grip force coordination on hand tools. Ergonomics. 1999;42(4):550–64. Epub 1999/04/16. 10.1080/001401399185469 . 10204420

[20] Nowak DA, Hermsdorfer J. Selective deficits of grip force control during object manipulation in patients with reduced sensibility of the grasping digits. Neurosci Res. 2003;47(1):65–72. . 12941448 10.1016/s0168-0102(03)00182-2

[21] Nunes PM, de Oliveira DG, Aruin AS, dos Santos MJ. Relationship between hand function and grip force control in women with hand osteoarthritis. J Rehabil Res Dev. 2012;49(6):855–65. Epub 2013/01/10. . 23299257

[22] Jerosch-Herold C, Shepstone L, Miller L, Chapman P. The responsiveness of sensibility and strength tests in patients undergoing carpal tunnel decompression. BMC Musculoskelet Disord. 2011;12:244 Epub 2011/10/29. 1471-2474-12-244 [pii] 10.1186/1471-2474-12-244 22032626 PMC3214851

[23] Viegas SF, Pollard A, Kaminksi K. Carpal arch alteration and related clinical status after endoscopic carpal tunnel release. J Hand Surg Am. 1992;17(6):1012–6. Epub 1992/11/01. . 1430926 10.1016/s0363-5023(09)91048-6

[24] Jerosch-Herold C. Assessment of sensibility after nerve injury and repair: a systematic review of evidence for validity, reliability and responsiveness of tests. J Hand Surg Br. 2005;30(3):252–64. Epub 2005/05/03. S0266-7681(04)00370-5 [pii] 10.1016/j.jhsb.2004.12.006 . 15862365

[25] Hsu HY, Kuo LC, Kuo YL, Chiu HY, Jou IM, Wu PT, et al Feasibility of a novel functional sensibility test as an assisted examination for determining precision pinch performance in patients with carpal tunnel syndrome. PloS One. 2013;8(8):e72064 Epub 2013/08/27. doi: 10.1371/journal.pone.0072064 PONE-D-12-37741 [pii]. 23977209 10.1371/journal.pone.0072064 PMC3748063

[26] Hsu HY, Kuo YL, Jou IM, Su FC, Chiu HY, Kuo LC. Diagnosis from Functional Perspectives: The Usefulness of a Manual Tactile Test for Predicting Precision Pinch Performance and Disease Severity in Subjects with Carpal Tunnel Syndrome. Arch Phys Med Rehabil. 2013. Epub 2013/12/21. S0003-9993(13)01237-9 [pii] 10.1016/j.apmr.2013.11.017 . 24355426

[27] Rajesh KS, Lowery LET. The Electromyographer’s Handbook. 2nd ed. Boston/Toronto: Little Brown and Company.; 1989.

[28] Chiu HY, Hsu HY, Kuo LC, Chang JH, Su FC. Functional sensibility assessment. Part I: develop a reliable apparatus to assess momentary pinch force control. J Orthop Res. 2009;27(8):1116–21. Epub 2009/02/06. 10.1002/jor.20859 . 19195027

[29] Voerman VF, van Egmond J, Crul BJ. Elevated detection thresholds for mechanical stimuli in chronic pain patients: support for a central mechanism. Arch Phys Med Rehabil. 2000;81(4):430–5. Epub 2000/04/18. S0003-9993(00)84149-0 [pii] 10.1053/mr.2000.3777 . 10768531

[30] Kadouri A, Corruble E, Falissard B. The improved Clinical Global Impression Scale (iCGI): development and validation in depression. BMC Psychiatry. 2007;7:7 Epub 2007/02/08. 1471-244X-7-7 [pii] 10.1186/1471-244X-7-7 17284321 PMC1802073

[31] Kazis LE, Anderson JJ, Meenan RF. Effect sizes for interpreting changes in health status. Med Care. 1989;27(3 Suppl):S178–89. Epub 1989/03/01. . 2646488 10.1097/00005650-198903001-00015

[32] Cohen J. Statistical power analysis for the behavioral sciences. 2nd ed. Hillsdale: Lawrence Erlbaum; 1988.

[33] Faul F, Erdfelder E, Lang AG, Buchner A. G*Power 3: a flexible statistical power analysis program for the social, behavioral, and biomedical sciences. Behav Res Methods. 2007;39(2):175–91. Epub 2007/08/19. . 17695343 10.3758/bf03193146

[34] Jerosch-Herold C. A study of the relative responsiveness of five sensibility tests for assessment of recovery after median nerve injury and repair. J Hand Surg Br. 2003;28(3):255–60. Epub 2003/06/18. S0266768103000172 [pii]. . 12809660 10.1016/s0266-7681(03)00017-2

[35] Benson LS, Bare AA, Nagle DJ, Harder VS, Williams CS, Visotsky JL. Complications of endoscopic and open carpal tunnel release. Arthroscopy. 2006;22(9):919–24, 24 e1-2. Epub 2006/09/06. S0749-8063(06)00693-1 [pii] 10.1016/j.arthro.2006.05.008 . 16952718

[36] Klatzky RL, Loomis JM, Lederman SJ, Wake H, Fujita N. Haptic identification of objects and their depictions. Percept Psychophys. 1993;54(2):170–8. Epub 1993/08/01. . 8361831 10.3758/bf03211752

[37] Hsu HY, Kuo LC, Jou IM, Chen SM, Chiu HY, Su FC. Establishment of a proper manual tactile test for hands with sensory deficits. Arch Phys Med Rehabil. 2013;94(3):451–8. Epub 2012/08/14. doi: 10.1016/j.apmr.2012.07.024 S0003-9993(12)00594-1 [pii]. . 22885285 10.1016/j.apmr.2012.07.024

[38] Craig JC, Johnson KO. The two-point threshold: not a measure of tactile spatial resolution. Current Directions in Psychological Science. 2000;9:29–32.

[39] Kimmerle M, Mainwaring L, Borenstein M. The functional repertoire of the hand and its application to assessment. Am J Occup Ther. 2003;57(5):489–98. Epub 2003/10/07. . 14527110 10.5014/ajot.57.5.489

[40] Witney AG, Wing A, Thonnard JL, Smith AM. The cutaneous contribution to adaptive precision grip. Trends Neurosci. 2004;27(10):637–43. . 15374677 10.1016/j.tins.2004.08.006

[41] Yen WJ, Kuo YL, Kuo LC, Chen SM, Kuan TS, Hsu HY. Precision pinch performance in patients with sensory deficits of the median nerve at the carpal tunnel. Motor Control. 2014;18(1):29–43. Epub 2014/02/06. 10.1123/mc.2013-0004 . 24496877

[42] Fitts PM. The information capacity of the human motor system in controlling the amplitude of movement. J Exp Psychol. 1954;47(6):381–91. Epub 1954/06/01. . 13174710

[43] Hsu HY, Kuo LC, Chiu HY, Jou IM, Su FC. Functional sensibility assessment. Part II: Effects of sensory improvement on precise pinch force modulation after transverse carpal tunnel release. J Orthop Res. 2009;27(11):1534–9. Epub 2009/04/30. 10.1002/jor.20903 . 19402148

[44] Shieh SJ, Hsu HY, Kuo LC, Su FC, Chiu HY. Correlation of digital sensibility and precision of pinch force modulation in patients with nerve repair. J Orthop Res. 2011;29(8):1210–5. Epub 2011/03/05. 10.1002/jor.21365 . 21374708

[45] Craig JC. Grating orientation as a measure of tactile spatial acuity. Somatosens Mot Res. 1999;16(3):197–206. Epub 1999/10/20. . 10527368 10.1080/08990229970456

[46] Van Boven RW, Johnson KO. The limit of tactile spatial resolution in humans: grating orientation discrimination at the lip, tongue, and finger. Neurology. 1994b;44(12):2361–6. Epub 1994/12/01. . 7991127 10.1212/wnl.44.12.2361

